# Evaluating the safety and feasibility of a new surgical treatment for forearm fractures in older children: study protocol for a randomised controlled trial

**DOI:** 10.1186/s13063-019-3458-5

**Published:** 2019-06-03

**Authors:** Chunhui Chen, Linzhen Xie, Wenhao Zheng, Hua Chen, Leyi Cai

**Affiliations:** Department of Orthopaedics, The Second Affiliated Hospital and Yuying Children’s Hospital of Wenzhou Medical University, 109 Xue Yuan Xi Road, Wenzhou, 325000 Zhejiang China

**Keywords:** Forearm fractures, Elastic stable intramedullary nailing, Hybrid fixation, Randomised controlled trial

## Abstract

**Background:**

Both-bone forearm fractures are a common fracture, accounting for 3.4% of all paediatric fractures. For now, elastic stable intramedullary nailing (ESIN) and open reduction and internal fixation (ORIF) are the common surgical procedures for paediatric both-bone forearm fractures. Both ORIF and ESIN have their shortcomings. Therefore, we need to find another surgical treatment which can decrease the rate of complications and improve the clinical efficacy. Our study plans to test hybrid fixation, using an ESIN fixation for the radius and an ORIF for the ulna. Our study will conduct a randomised controlled trial (RCT) comparing double plate fixation with hybrid fixation for treatment of both-bone forearm fractures in older children between 10 and 16 years of age. The objectives of this trial are to compare the effectiveness between double plate fixation and hybrid fixation for treatment of both-bone forearm fractures in older children.

**Methods:**

An RCT will be conducted, and the participants included will be randomly divided into either the hybrid fixation group or the double plate fixation group, at a ratio of 1:1. The primary clinical outcome measures are the Disabilities of the Arm, Shoulder and Hand score and radiological evaluation. Secondary clinical outcome measures are intraoperative blood loss, surgical duration, visual analogue scale score after surgery, hospital duration after surgery and complications. Follow-up will be conducted at 2 weeks and 1, 3, 6 and 12 months postoperatively.

**Discussion:**

The trial will provide a new surgical treatment for forearm fractures in older children. Our hypothesis is that there is no clinically relevant difference in the primary outcome measures between the two treatment groups.

**Trial registration:**

Chinese Clinical Trial Registry, ChiCTR1800018060. Registered on 26 August 2018.

**Electronic supplementary material:**

The online version of this article (10.1186/s13063-019-3458-5) contains supplementary material, which is available to authorized users.

## Background

Both-bone forearm fractures are a common fracture, accounting for 3.4% of all paediatric fractures and 26% of paediatric upper extremity fractures [1, 2]. For children under 10 years old, most both-bone forearm fractures can be treated successfully with casting due to the considerable bone remodelling potential [3–5]. Although closed reduction and casting is a feasible choice for children older than 10 years old [6], there are adverse outcomes of conservative treatment, such as nonunion of the fracture, malunion and secondary surgery [7]. Also, the standard of amount of angulation or malrotation is not clear in this age group. In addition, for open fractures, fractures associated with compartment syndromes, elbow injuries, combined injuries such as Monteggia fractures and Monteggia equivalents, significant comminution or further displacement with nonoperative treatment, operative treatment is required [8–10]. For now, elastic stable intramedullary nailing (ESIN) and plate screw fixation are the common surgical procedures for paediatric both-bone forearm fractures [11]. The advantages of ESIN fixation for paediatric both-bone forearm fractures compared with plate fixation include less wound infection, shorter operative time, smaller edge, less soft tissue dissection, ease of implant removal and early return to activity after implant removal [12–16]. However, ESIN fixation also has its own shortcomings, including delayed union and nonunion, refracture, implant migration or failure and compartment syndrome [17–21]. Compared with younger children, the rate of complications is obviously increased in those over 10 years old [22–24]. Open reduction and internal fixation (ORIF) is a surgical alternative in this age group and offers some potential benefits, including immediate fracture stabilisation and anatomic reduction, which are important for restoring forearm rotation [25, 26]. However, ORIF has been criticised because of its longer operation time, the amount of soft tissue dissection and periosteal stripping, the increased amount of bleeding and the increased risk of wound infection [27, 28].

Both ORIF and ESIN fixation have their shortcomings. Therefore, we need to find another surgical treatment which can decrease the rate of complications and improve the clinical efficacy. Our study plans to test hybrid fixation, using an ESIN fixation for the radius, and open reduction and plate screw fixation for the ulna. Compared with double plate fixation, hybrid fixation not only reduces soft tissue dissection and potentially refracture rates after implant removal, but it also incorporates some advantages of ESIN fixation. Feng et al. conducted a case-control study and found that mixed fixation was a feasible method [29]. Our study will conduct a randomised controlled trial (RCT) comparing double plate fixation with hybrid fixation for treatment of both-bone forearm fractures in older children between 10 and 16 years of age.

The objectives of this trial are to compare the effectiveness between double plate fixation and hybrid fixation for treatment of both-bone forearm fractures in older children. Our hypothesis is that there is no clinically relevant difference in the primary outcome measures between the two treatment groups.

## Methods

### Study setting

**Fig. 1:**
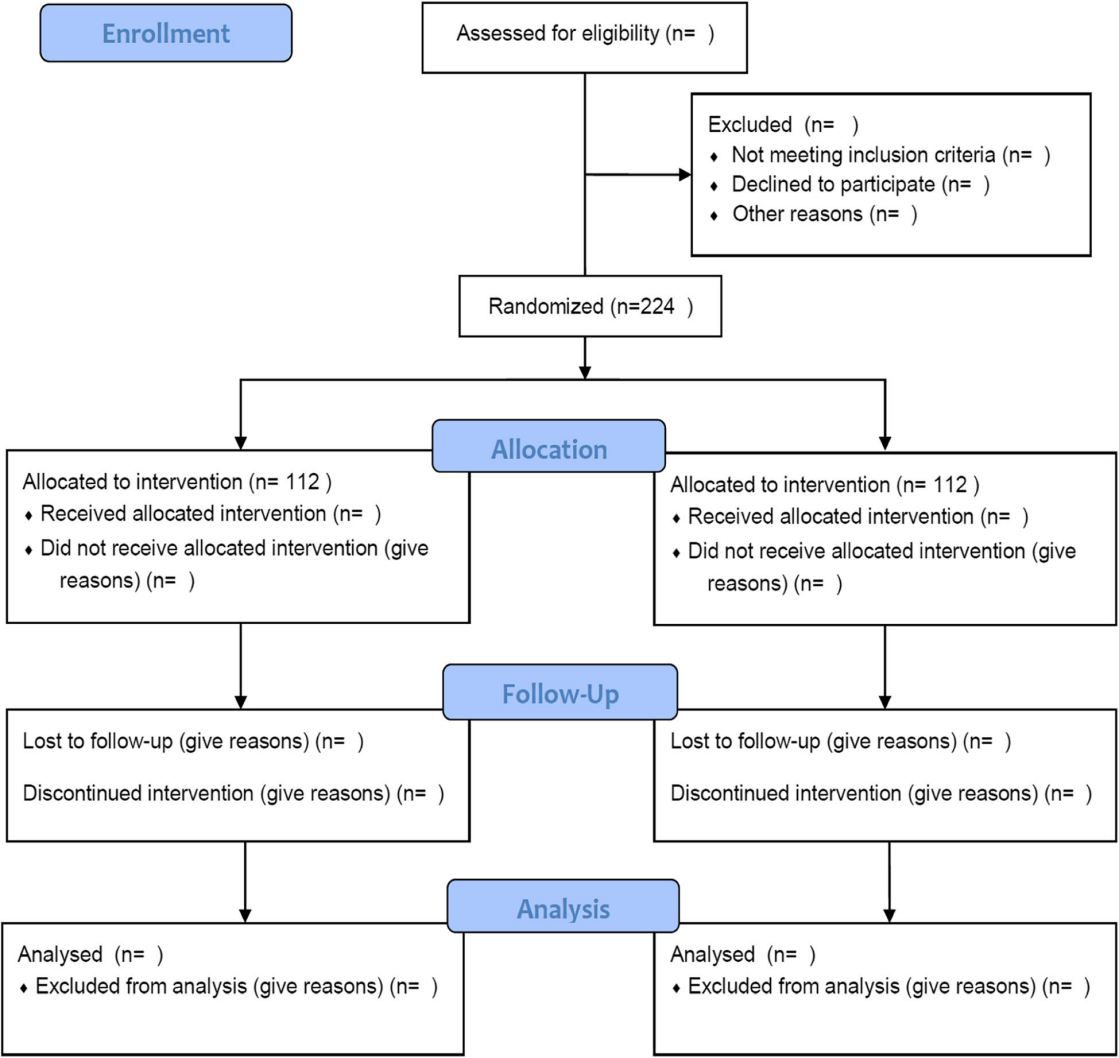
Flow chart of the trial

The study, based on an RCT, will be conducted in our hospital. The trial was approved and monitored by the Ethics Research Committee of our hospital, and it conforms to the Declaration of Helsinki Ethical Principles for Medical Research Involving Human Subjects. This trial has been registered at the Chinese Clinical Trial Registry (ChiCTR1800018060). The protocol conforms to the Standard Protocol Items: Recommendations for Interventional Trials (SPIRIT) guidelines (see Additional file 1). Figure 1 shows a flow chart of the trial design.

### Consent

Informed consent takes place in a face-to-face setting at the research site. Patients’ parents will have at least 24 h to consider participation and will be encouraged to discuss the study with their family and other healthcare professionals. A full verbal explanation of the study, a written Patient Information Sheet (detailing rationale, design and personal implications of trial entry) and informed consent form will be provided. Participants may withdraw at any stage of the trial. Consent will be obtained prior to collection of baseline assessment data and subsequent randomisation.

### Participants

Some members of our group will assess patients with both-bone forearm fractures for eligibility. The diagnosis will be verified using anteroposterior and laterolateral radiographs. All eligible patients’ parents will be introduced to the study, given detailed written information about it and then asked to participate and to sign the written informed consent form. Inclusion and exclusion criteria are listed as follows.

#### Inclusion criteria

The inclusion criteria are as follows:

1. Boys or girls aged 10–16 years old
2. Only unilateral displaced closed both-bone forearm fractures
3. AO fracture classification types 22-A3 and 22-B3
4. Fracture has been present for less than 10 days
5. The time from injury to operation is less than 14 days
6. Parents have signed informed consent form and are willing to participate in all follow-up visits.

#### Exclusion criteria

The exclusion criteria are:

1. Bilateral fracture, open fractures, complex forearm fractures (Monteggia fractures, Galeazzi fractures, intra-articular elbow or wrist fractures) and pathologic fractures
2. History of trauma of the same upper extremity causing functional deficit
3. Disease that significantly affects the general condition of the patient
4. Significantly impaired ability to cooperate for any reason (substance abuse, mental disorder, dementia)
5. Unwilling to accept both treatment methods.

### Participant withdrawal criteria

Patients will be withdrawn for the trial for the following reasons:

1. Patients request to withdraw from the trial
2. Occurrence of a serious adverse event
3. Occurrence of factors making it difficult to sustain the process or investigator’s decision to terminate because of clinical trial results affected by some factors
4. Patient death or patient lost to follow-up.

### Participant timeline

**Fig. 2:**
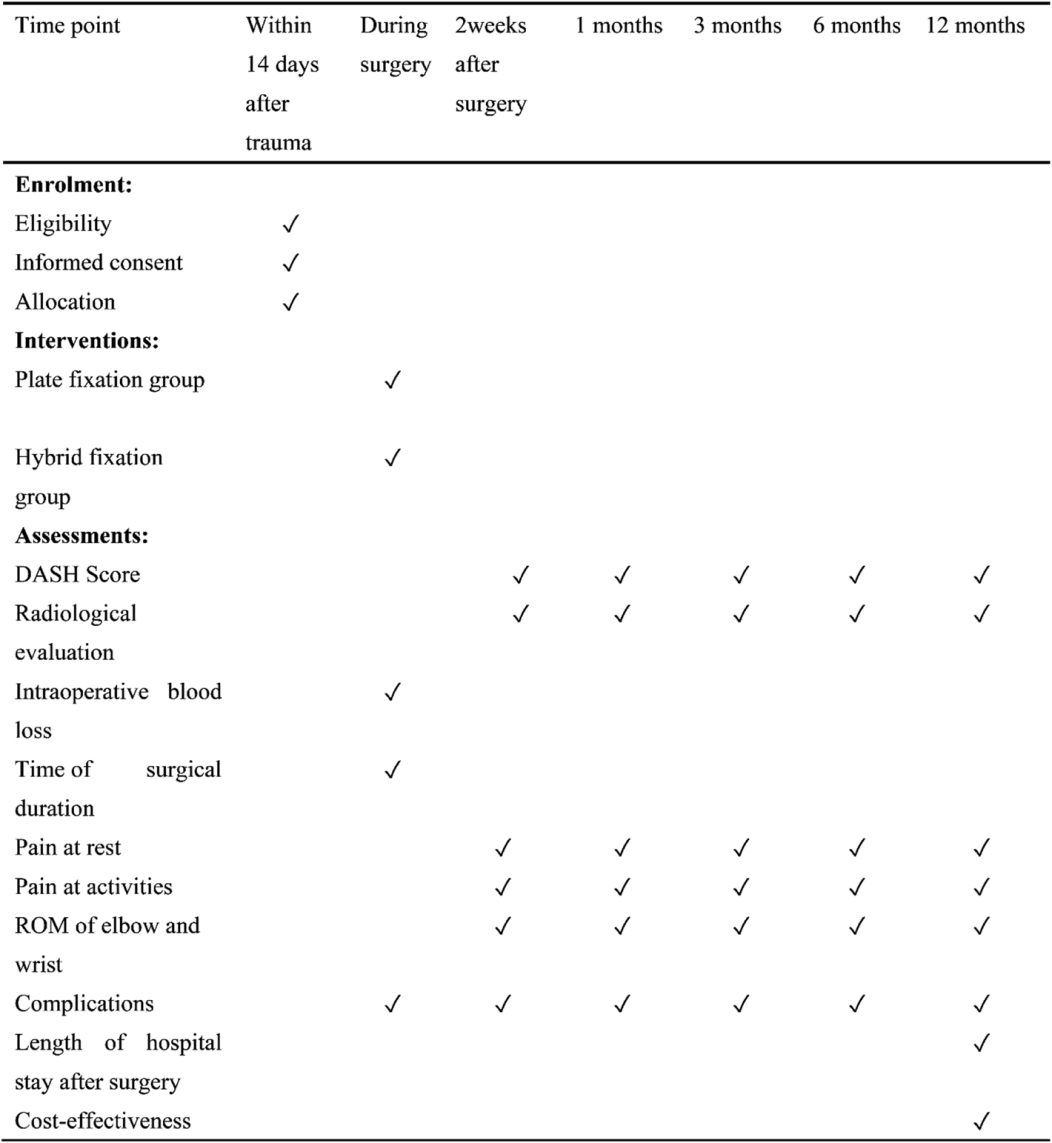
Schedule of enrolment, interventions and assessments

A flow chart of the trial is presented in Fig. 1. The time schedule of enrolment, interventions, assessments and visits is shown in Fig. 2.

### Sample size

The sample size calculation is performed using G*Power 3.1 [30] and is based on Disabilities of the Arm, Shoulder and Hand (DASH) score as the primary outcome measure in this trial. For the sample size calculation, we used an α level of 0.05 and a β level of 0.1. We assumed the minimal clinically important difference (MCID) of the DASH to be 5 points, with the standard deviation (SD) being 14.7 [31]. Using these assumptions, the required sample size is 98 per group with 90% power to show a clinically important difference between the treatment methods with a two-sided type I error rate of 5%. With an assumption of 12.5% lost to follow-up, we decided to include 112 participants per group.

### Allocation and randomisation

Prerandomisation eligibility checks will be carried out to ensure that participants are eligible for inclusion in the study. Patients will be randomly assigned to one of two groups (experimental or control) using a computer-generated random assignment in a 1:1 ratio. The computer randomly extracts 112 numbers from the 1–224 number as the hybrid fixation group, and the remaining numbers as the plate fixation group. According to the order of inclusion, patients will be numbered 1–224. Patients, researchers performing the follow-up measurements and the trial statistician will be blinded to the group allocations.

### Blinding

Blinding in this study is almost impossible. The surgical approach differs considerably between these two methods. All patients, researchers and surgeons can also see the differences in the skin incisions between the ESIN and ORIF treatments.

### Interventions

Surgical treatment will be performed either by or under the supervision of an experienced orthopaedic surgeon within 2 weeks after initial trauma.

#### Plate fixation group

A 3.5-mm narrow locking compression plate (DePuy Synthes, Raynham, MA, USA) will be the preferred choice of the treating surgeon. The length of the plate will be at least 7 holes to ensure stability of osteosynthesis, and at least three bicortical screws will be used on both sides of the fracture line. For dual plating fixation constructs, ORIF of the ulna will be performed using the direct approach to the subcutaneous ulnar shaft. The radius will be exposed with a standard anterior (Henry) approach. The dynamic compression plates will be used for both the radius and ulna. Reduction will be obtained and provisionally fixated; fluoroscopic image intensification will be used to verify rotational alignment and reduction of both fractures. Fixation of the implants will then be definitively secured.

#### Hybrid fixation group

The Titanium Elastic Nail System from Synthes (DePuy Synthes) with ESIN nails of 2.0–3.0 mm diameter, appearing to be one third of the diameter of the central bony canal, are usually used in our Department of Trauma Surgery. For hybrid constructs, the patient will be placed on the table and an image intensifier will be used to localise the placement of skin incisions. The radial ESIN nail is inserted through a 1- to 2-cm mini incision, to protect the superficial radial nerve, at the distal lateral radius. When the nail reaches the fracture site, the fracture is reduced by manipulation and traction under image intensifier control. Once reduction, alignment and provisional fixation of both fractures is satisfactory, the straight rod will be removed and an appropriately sized elastic intramedullary nail will be inserted into the radius. The plate on the ulnar will then be definitively affixed to the bone. An above-elbow plaster cast will be applied and maintained for 2 weeks.

### Outcomes

Clinical radiological evaluation of union, functional evaluation of outcome and rate of complications will be performed immediately, and at 2 weeks and 1, 3, 6 and 12 months.

#### Primary outcome measures

Primary outcome measures are DASH score and radiological evaluation:

1. The primary outcome measure of this study is the DASH score, which will be recorded at 1, 3, 6 and 12 months postoperatively. The primary time point is at 12 months. DASH is a widely used and validated tool assessing upper extremity-related deficits and symptoms in daily life reported by the patient. It has been shown to be a valid instrument to monitor changes in symptoms and function over time [32–34].
2. Radiological evaluation based on postoperative X-ray, including nonunion of the fracture and malunion of the fracture, will be performed at 2 weeks and 1, 3, 6 and 12 months postoperatively.

#### Secondary outcome measures

Measurements are recorded at 2 weeks and 1, 3, 6 and 12 months postoperatively.

1. Intraoperative blood loss will be recorded in the anaesthesia records and will include the blood in suction bottles (after subtracting the lavage fluid used during the surgery) and that in the weighed sponges used during the operation.
2. Time of surgical duration.
3. Pain at rest (0–10 score with visual analogue scale).
4. Pain at activities (0–10 score with visual analogue scale).
5. Range of motion (ROM) of elbow and wrist.
6. Complications: wound infection, reoperations and nerve injury.
7. Length of hospital stay after surgery.
8. Cost-effectiveness.

### Data collection and management

Questionnaire forms on paper will be the primary data collection tools for the study. The questionnaires will be completed at the outpatient clinic during the baseline and control visits. On receipt of the questionnaire forms, the researcher will make a visual check of the responses and will query missing data when possible. The paper forms will be securely stored at both study sites. Double data entry will be used to minimise typing errors. A research nurse and a research assistant will enter the data independently into two separate electronic databases. First, the research nurse enters the data into an electronic database, which is located in a secure network drive and protected with access codes known only by the research nurse. Missing, implausible and inconsistent data in the electronic database will be checked by the research nurse at the coordinating centre. If a missing or implausible item is noticed, the patient will be contacted and asked about the item. The answer will be corrected on the original paper form with a note that the answer was retrieved by a phone call and the corrected data had been entered to the database.

Patient records in the participating hospitals will also be used when collecting missing data or interpreting inconsistent or implausible data. After 12 months follow-up visits are completed and all data stored, a research assistant, not involved in the trial, will enter all the data from the paper forms into a separate database. The two databases will be compared for consistency. Discrepancies will be checked from the original paper forms by a research nurse at the coordinating centre. Final interpretation of the data will be corrected into the master database, which will be the source for the final data analysis.

### Monitoring

#### Data monitoring

We will conduct the study without a data monitoring committee (DMC). Both treatment methods are widely used in daily practice and have been proven to provide acceptable results. Since there is no DMC, we will not conduct an interim analysis during the trial.

#### Harms auditing

All the medical records of the participating patients will be carefully assessed, and all harms and complications of the treatment will be reported when reporting the results of this trial. The harms will be categorised as serious and minor adverse events as described in the section.

#### Auditing

We will not conduct auditing between the participant centres during the trial.

### Follow-up

Follow-up will be conducted at 2 weeks and 1, 3, 6 and 12 months postoperatively.

### Statistical analysis

The trial data will be analysed using SPSS for Windows software (V.19.0; SPSS, Chicago, IL, USA). For continuous variables, the Shapiro-Wilk test will be applied to determine if they follow a normal distribution. For normally distributed variables, the means will be calculated and compared using the independent samples *t* test (Student’s *t* test) or analysis of variance (ANOVA); otherwise, the Mann-Whitney *U* test will be used for group comparisons. The χ2 test will be used to analyse qualitative variables. In all analyses, *p* < 0.05 will be taken to indicate statistical significance.

### Ethics and dissemination

#### Research ethics approval

The trial was approved and monitored by the Ethics Research Committee of our hospital, which conforms to the Declaration of Helsinki Ethical Principles for Medical Research Involving Human Subjects. This trial has been registered at the Chinese Clinical Trial Registry (ChiCTR1800018060). The protocol conforms to the Standard Protocol Items: Recommendations for Interventional Trials.

#### Protocol amendments

All modifications of the study protocol will be communicated by updating the trial registry (Chinese Clinical Trial Registry.gov).

#### Dissemination policy

The findings of this study will be disseminated through peer-reviewed publications and conference presentations. Patients participating in the trial will be sent a letter with information on the results after the primary outcome results are published.

## Discussion

### Potential impact and significance of the study

ORIF treatment of forearm double fracture requires two longer surgical incisions to achieve satisfactory fracture reduction and fixation. To obtain adequate exposure, a significant amount of soft tissue dissection and periosteal stripping may be necessary, which has been regarded as a risk factor for nonunions [35, 36]. Compared with ORIF, ESIN has the advantage of smaller incisions, shorter operative time and less bleeding in the treatment of forearm fractures. However, this method may lead to increased redisplacement and reduced clinical results, which may fail to provide rotational stability [16]. Both ORIF and ESIN fixation have their shortcomings. Therefore, we need to find another surgical treatment which can decrease the rate of complications and improve the clinical efficacy.

The purpose of our study is to evaluate hybrid fixation construct feasibility, using an ESIN fixation for the radius and open reduction and plate screw fixation for the ulna.

The titanium elastic nail is known for its good flexibility with antirotation performance to some extent, which can be remodelled according to the curvature of the radius. It can also be easily prebent to make a fixation with two or more points in accordance with the fracture characteristics and location. Plate fixation of the ulna makes the forearm more stable, further enhancing the antirotation performance. Thus, patients do not need a long-time plaster cast applied, which is conducive to early exercise. Salvi [37] considered that the radius and ulna meet different functions; the radius had more complex functions, such as pronation and supination, whereas the ulna played a greater role in maintaining the stability of the forearm with respect to the radius, especially when subjected to buckling and torsional stress. So, when the ulna was intact, the radius fractures treated using intramedullary fixation would achieve a greater antirotation performance [38]. Therefore, restoring the original function of the ulna was necessary to rebuild the forearm stability, and ulnar plate fixation achieved this goal precisely.

In this protocol paper we describe an RCT comparing plate fixation with hybrid fixation for treatment of both-bone forearm fractures in older children between 10 and 16 years of age. After completion, this trial will provide valuable evidence on the treatment of both-bone forearm fractures in older children.

### Strengths and limitations of the study

This study has potential limitations. Participants are under 16 years old, and their responses might not really reflect their subjective feelings. Thus, certain external validity problems will remain as a significant proportion of these patients are noncooperative. In addition, our follow-up period is only 1 year, because of limited funds and researchers.

### Expectations

Our expectation is that there will be no clinically relevant difference in the primary outcome measure between the two treatment groups. Hybrid fixation will be another effective option for treatment of both-bone forearm fractures in older children.

### Trial status

This trial is ongoing, and patient recruitment began in October 2018. Recruitment is expected to be completed in October 2019.

## Additional file

Additional file 1: SPIRIT 2013 checklist: recommended items to address in a clinical trial protocol and related documents. (DOC 124 kb)

## Data Availability

The data that support the findings of this study will be available from Wenzhou Medical University; however, restrictions apply to the availability of these data. The data will be used under a license published for the current study and will not be publicly available. However, the data will be available from the corresponding author upon reasonable request and with permission from the institutions.

## Abbreviations

ChiCTR: Chinese Clinical Trial Registry
DASH: Disabilities of the Arm, Shoulder and Hand
ESIN: Elastic stable intramedullary nailing
MCID: Minimal clinically important difference
ORIF: Open reduction and plate screw fixation
RCT: Randomised controlled trial
ROM: Range of motion
